# CD73 Severed as a Potential Prognostic Marker and Promote Lung Cancer Cells Migration *via* Enhancing EMT Progression

**DOI:** 10.3389/fgene.2021.728200

**Published:** 2021-11-17

**Authors:** Zhao-wei Gao, Chong Liu, Lan Yang, Hao-chuan Chen, Long-fei Yang, Hui-zhong Zhang, Ke Dong

**Affiliations:** Department of Clinical Laboratory, The Second Affiliated Hospital, Air Force Medical University, Xi’an, China

**Keywords:** lung cancer, CD73, migration, epithelial to mesenchymal transition, progonosis

## Abstract

To investigate the expression levels and prognostic value of CD73 in lung cancer. And moreover, to identify the effect and potential mechanism of CD73 on lung cancer cells proliferation and migration. CD73 expression levels in lung cancer were analyzed base on GEPIA2 and GEO database. GEPIA2 and Kaplan-Meier Plotter (KM Plotter) was used to analyzed the correlation between CD73 expression and prognosis. GEO dataset were analyzed via GEO2R. CD73 overexpression cell model was construction via recombinant lentivirus transfection into A549 and NCI-H520 cells. CCK8 assay were used to investigate cells proliferation. Migration and invasion ability were evaluated by scratch and transwell methods. Base on GEPIA2, GSE32683, GSE116959 and GSE37745 dataset, we found that CD73 expression were significant higher in tumor tissues of lung adenocarcinoma (LUAD) compared with that in non-tumor normal tissues and in lung squamous cell carcinoma (LUSC), while there were no significant difference of CD73 expression between LUSC and normal control tissues. Interestingly, a high CD73 level predict poor overall survival (OS) of LUSC. However, GEPIA2 and KM plotter showed the opposite conclusion of prognostic value of CD73 in LUAD. By using cell experiments, we found that CD73 overexpression promoted proliferation and migration of LUAD A549 cells. However, there was no significant effect of CD73 overexpression on LUSC NCI-H520 cells. Furthermore, CD73 overexpression facilitates epithelial to mesenchymal transition (EMT) progression of A549 cells. In conclusion, our results indicated that CD73 expression were increased in LUAD and might be an poor prognostic marker for LUSC patients. CD73 play an important role in LUAD cells proliferation and migration. These data allowed to support CD73 as a therapeutic target for LUAD.

## Introduction

Lung cancer is the most common cancer type in the world, with the highest morbidity and mortality (Siegel et al., 2019). Among lung cancer patients, lung adenocarcinoma (LUAD) and lung squamous cell carcinoma (LUSC) were are the two most common subtypes (Testa et al., 2018). Metastasis is an important cause of death in lung cancer patients. However, the molecular mechanism of lung cancer metastasis is complex and has not been fully elucidated.

CD73 is a glycosylphosphatidylinositol-anchored cell surface protein, which is encoding by NT5E gene (ID: 4907) (Zimmermann et al., 2012). As the ecto-5′-nucleotidase (EC 3.1.3.5), CD73 catalyzes the degradation of adenosine, which acts as an immuno-suppression signal and play an important role in immuno-regulation (Boison and Yegutkin, 2019; Allard et al., 2020). In addition, CD73 also acts as an non-enzymatic molecular, which was involved in cell’s proliferation, migration and adhesion (Sadej and Skladanowski, 2012; Gao et al., 2014). Expression levels of CD73 were changed in a variety of tumors, and play an important role in tumor genesis, metastasis and drug resistance (Turcotte et al., 2017; Turiello et al., 2020; Yang et al., 2021).

In this present study, we first analyzed the differential CD73 expression in lung cancer tumor tissues compared to non-tumor normal tissues. And also analyzed the potential prognostic value of CD73 expression in lung cancer. And furthermore, we investigated the effect and mechanism of CD73 overexpression in biological behavious of lung cancer A549 cells. This study may present evidences of CD73 as a potential therapeutic target for lung cancer, especially for LUAD.

## Materials and Method

### Expression Data Analysis of CD73

By using GEPIA2, differential expression between LUAD or LUSC and normal controls were performed with the option of matching TCGA normal and GTEx data (Tomczak et al., 2015; Tang et al., 2017). GSE32683 and GSE116959 datasets were used to evaluate the difference of CD73 expression between LUAD tumor tissue and non-tumor tissue (Selamat et al., 2012; Moreno Leon et al., 2019). GSE32683 dataset contains gene expression profiling of 60 LUAD and matched adjacent non-tumor lung tissue. GSE116959 dataset contains profiling of 57 LUAD samples and 11 peritumoral normal lung tissues. GSE37745 dataset were used to analyzed the difference of CD73 expression between LUAD and LUSC (Botling et al., 2013; Lohr et al., 2015; Jabs et al., 2017). GSE31552 and GSE103512 datasets were used to analyzed the difference of CD73 expression between LUSC and normal controls (Lin et al., 2014; Brouwer-Visser et al., 2018).

### Prognostic Value Analysis of CD73

GEPIA2 and KM plotter (Menyhárt et al., 2018) were used to evaluate the prognostic value of CD73 in LUAD and LUSC. For GEPIA2, the source data was based on TCGA database, we choose a median expression as cut-off for splitting the high-expression and low-expression cohorts. For KM plotter, the source data was based on GEO datasets (GSE19188, GSE29013, GSE30219, GSE31210, GSE3141, GSE37745, GSE50081), all possible cut-off values between the lower and upper quartiles are computed, and the best performing threshold is used as a cut-off.

### Cell Culture and CD73 Overexpression Cell Model Construction

LUAD cell line-A549 and LUSC cell line-NCI-H520 cells were cultured with DMEM plus 10% FBS (SiJiQing, Hangzhou, China) in 5% CO_2_ incubator at 37°C. CD73 overexpression recombinant lentivirus (Genechem, Shanghai, China) were transfected into A549 and NCI-H520 cells according to manufacturer’s instructions. Stable cell models were screened with 2.5 μg/ml puromycin, named as A549-CD73 and NCI-H520-CD73. Reverse transcription-quantitative PCR and Western-blot were used to evaluate the CD73 expression level in cell models. 100μM APCP (Sigma-Aldrich) treatment were used to inhibit the CD73 enzymatic activity in A549-CD73 cells.

### Cell Counting Kit-8 (CCK8) Assay

The cells were seeded in 96 well plate at a density of 2 × 10^3^ cells per well. Cell proliferation were detected by CCK8 assay according to manufacturer’s instructions (KeyGENBiotech, Nanjing, China). In short, at different time point, 10 μl of CCK8 reagent was added into each well and the cells were incubated for 2 h at 37°C. Then, the absorbance value was measured at 450 nm by using the microplate reader (Epoch, BIO-TEK). The relative viability of cells was calculated as a percentage using the formula: (mean OD450 of treated cells/mean OD450 of control cells) × 100%.

### Colony Formation Assay

For the colony formation assay, 200 cells were seeded in 9 cm culture dishes, and After 10 days incubation at 37°C with 5% CO2 in a humidified incubator, the cells were fixed with methanol and stained with crystal violet. Visible colonies were counted.

### Cell Migration Assay

As previously described (Gao et al., 2016), cell’s migration ability of was examined by scratch assay. In brief, cells were seeded and cultured in 6-well plate. The scratch was performed when cell density reached to 80%. Then, cells were cultured with serum-free DMEM. The scratch image were captured at 0 and 24 h. Cell migration ratio = (start distant - end distant)/start distant.

### Invasion Assay

As previously described (Gao et al., 2016), invasion assay was performed in transwell chamber. Cells were seeded in matrigel coated filters, 200 μl DMEM were added to upper compartments of the chambers while 500 μl DMEM plus 10% FBS was added to lower compartments. After 24 h incubation, the cells on the upper surface of the filter was wiped off, then, the cells on the lower surface of the filters were fixed with ethanol, stained with crystal violet and counted.

### Quantitative Real-Time RT-PCR

Quantitative real-time RT-PCR (qRT-PCR) was carried out using FastStart Essential DNA Green Master kit (Roche) and was used to detect the mRNA expression levels. The primer sequences were list in Table 1.

**TABLE 1:** Primer sequence used in qRT-PCR analysis.

Gene	Forward primer (5–3′)	Reverse primer (5–3′)
CD73	GCC​TGG​GAG​CTT​ACG​ATT​TTG	TAG​TGC​CCT​GGT​ACT​GGT​CG
E-cadherin	TGC​CCA​GAA​AAT​GAA​AAA​GG	GTG​TAT​GTG​GCA​ATG​CGT​TC
Vimentin	CAT​CGA​CAA​GGT​GCG​CTT​CC	CCT​CGG​CCA​GGT​TGT​CGC​GC
N-cadherin	ATG​ACA​ATC​CTC​CAG​AGT​TTA	ATC​CTT​ATC​GGT​CAC​AGT​TAG
FN1	CGA​GAG​TAA​ACC​TGA​AGC​TG	CCT​TCA​TGG​CAG​CGG​TTT​GC
β-Actin	TGA​CGT​GGA​CAT​CCG​CAA​AG	CTG​GAA​GGT​GGA​CAG​CGA​GG

### Western-Blot Assay

Cell protein was separation with SDS-PAGE and transferred onto nitrocellulose membrane. Membranes were blocked for 2–3 h with 5% non-fat dried milk at room temperature, and then incubated with primary antibody (anti-CD73 mAb was purchased from BBI Life sciences; mAb of anti-E-Cadherin, anti-N-cadherin, anti-Flbronectin1were purchased from R&D Systems; anti-Vimentin mAb were purchased from Abcam) overnight at 4°C. After washed with TBST for three times (15 min each time), membranes were incubated with secondary antibody for 2 h. Proteins were detected with western blotting luminol reagent (Bio-Rad), β-actin or GAPDH was used as the internal standard.

### Cell Cycle Analysis

Cells were treated by using cell cycle detection kit (KeyGENBiotech, Nanjing, China) according to the manufacturer’s instructions. In short, cells were fixed by ethanol and stained with propidium iodide (PI) in buffer containing 10 μg RNase A. Cell cycle distribution was assessed by using FACS calibur flow cytometer (BD Biosciences).

### Statistical Analysis

CD73 expression in tissues were obtained from GEPIA2 and GEO database. Survival curves were obtained from GEPIA2 and KM plotter. The difference of CD73 expression value were analyzed by using t-test. All survival results are displayed with p-values obtained using the log-rank test. The data from cell experiments are expressed as the mean ± standard error. Data comparisons were conducted by using the Student’s t-test. *p* < 0.05 was considered to be statistically significant.

## Results

### CD73 Expression Levels Were Increased in LUAD

**FIGURE 1:**
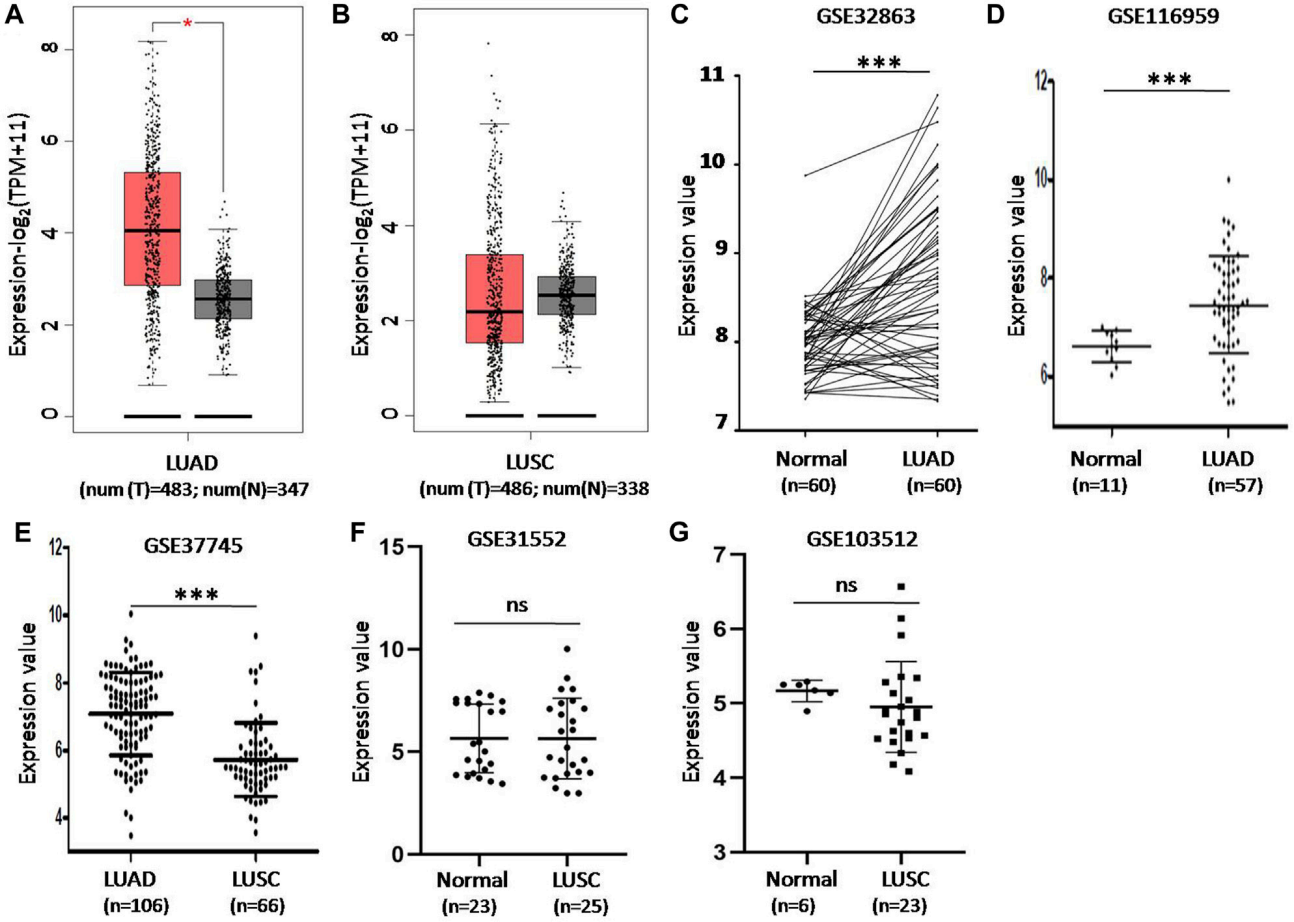
The difference of CD73 expression levels in lung cancer tumor tissue and non-tumor normal lung tissue in GEPIA2 and GEO database. **(A)** CD73 mRNA expression increased in LUAD tumor tissues. TPM: Transcripts Per Kilobase of exon model per Million mapped reads. **(B)** There were no significant difference of CD73 expression between LUSC tumor tissues and non-tumor normal tissues. **(C)** Differential expression of CD73 in GSE32683 dataset. **(D)** Differential expression of CD73 in GSE116959 dataset. **(E)** Differential expression of CD73 in GSE37745 dataset. **(F,G)** Differential expression of CD73 in GSE31552 and GSE103512 datasets * <0.05; ** <0.01; *** <0.001.

We first analyzed the expression levels of CD73 in LUAD and LUSC based on TCGA data via GEPIA2. Compared with that in normal tissues, the expression levels of CD73 were significant higher in tumor tissues of LUAD (Figure 1A). While there were no significant difference of CD73 expression between LUSC tumor and normal control tissues (Figure 1B).

CD73 expression levels in LUAD were also confirmed based on GSE32863 (gene expression profiling of 60 LUAD and 60 matched adjacent non-tumor lung tissues) and GSE11659 dataset (transcriptome profiling of 57 LUAD samples and 11 peritumoral normal lung tissues). The results further demonstrated that CD73 were overexpression in LUAD tumor tissues (Figures 1C,D). Notably, GSE37745 dataset analysis showed that CD73 expression levels were higher in LUAD than that in LUSC tumor tissues (Figure 1E). And moreover, GSE31552 and GSE103512 datasets showed that there were no significant difference of CD73 expression level between LUSC and normal controls (Figures 1F,G).

### The Potential Prognostic Value of CD73 in LUAD and LUSC

**FIGURE 2:**
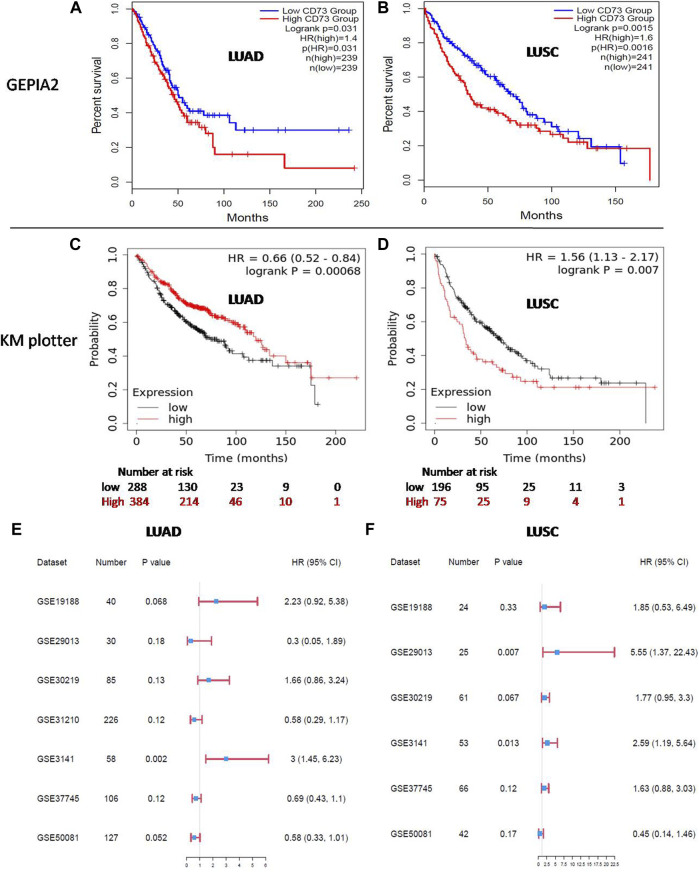
Prognosis of CD73 in LUAD and LUSC. **(A,B)** Kaplan-Meier (KM) survival analysis of CD73 in LUAD and LUSC via GEPIA2. **(C,D)** KM survival analysis of CD73 in LUAD and LUSC via KM plotter. **(E)** Forest plots showed the relation between CD73 expression and LUAD patients OS in different GEO datasets. **(F)** Forest plots showed the relation between CD73 expression and LUSC patients OS in different GEO datasets.

The prognostic value of CD73 in patients with LUAD and LUSC was analyzed by GEPIA2 and KM plotter. In GEPIA2, the results showed that a higher CD73 expression level was correlated with poor overall survival-OS in both LUAD [HR:1.4; logrank *p* = 0.031] (Figure 2A) and LUSC [HR: 1.5; logrank *p* = 0.018] (Figure 2B). However, in KM plotter, the results showed that a high CD73 expression correlated with better OS in LUAD (HR: 0.66; logrank *p* < 0.001) (Figure 2C), while correlated with poor OS in LUSC (HR: 1.56; logrank *p* = 0.007) (Figure 2D). These results indicated a significant association between high CD73 expression and poor prognosis of LUSC. Notably, the conflicting observation for prognostic significance of CD73 in LUAD, might be due to the different source data for GEPIA2 (TCGA database) and KM plotter (combined cohort of GEO datasets).

Furthermore, we analyzed the association of CD73 with patients OS in different GEO datasets separately. For LUAD (Figure 2E; Supplementary Figure S1A), high CD73 expression was related to a better OS (HR < 1) in four datasets, while high CD73 was related to a poor OS (HR > 1) in three datasets (HR > 1). Notably, among these datasets, the correlation between CD73 and OS with statistical significance were only found in GSE3141 (*p* = 0.002), While there were no statistical significance in other datasets (*p* > 0.05). For LUSC (Figure 2F; Supplementary Figure S1B), most of the GEO datasets (five in six) showed high CD73 as a poor prognostic marker (HR > 1), which were agreement in the results of GEPIA2 and KM plotter. Taken together, further verification for the potential prognostic value of CD73 in LUAD is needed.

### CD73 Overexpression Promote LUAD Cells Proliferation and Migration

**FIGURE 3:**
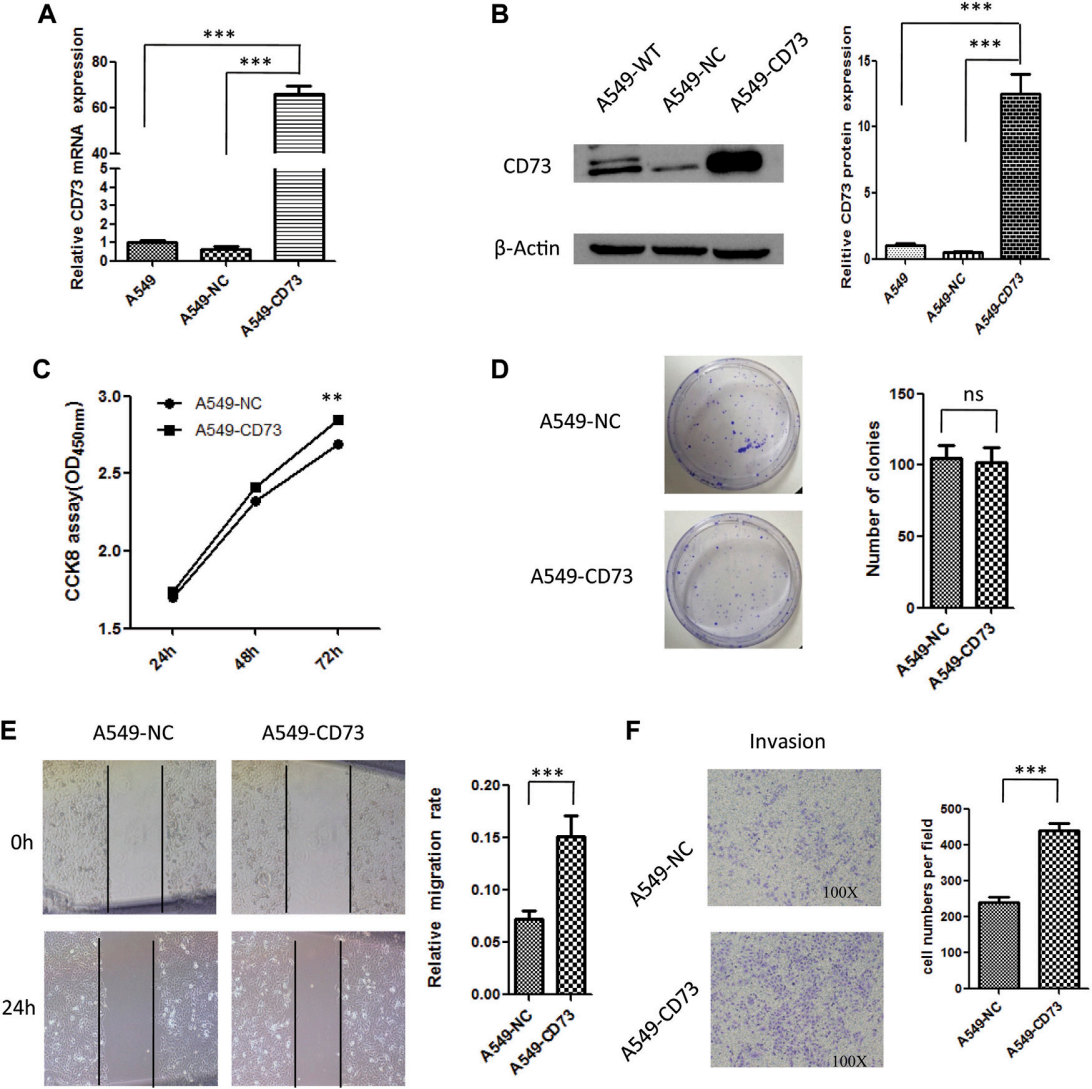
The effect of CD73 overexpression on biological behaviour of A549 cells. **(A)** CD73 mRNA expression levels were detected by q-RT-PCR. **(B)** CD73 protein expression levels were detected by western-blot. **(C)** The effect of CD73 overexpression on cells proliferation. **(D)** The effect of CD73 on colony formation. **(E)** The effect of CD73 on cells migration. **(F)** The effect of CD73 on cells invasion. ns: no significant difference; **p < 0.01; ***p < 0.001.

To further investigate the effect of CD73 on biological behavior of LUAD cancer cells. CD73 overexpression cell model was constructed and identified by q-RT-PCR and western-blot, which named as A549-CD73 (Figures 3A,B). By using CCK8 assay, we found that proliferation rate of A549-CD73 were significant higher than that of control cells (Figure 3C). While there were no significant difference of colony formation ability between A549-CD73 and control (Figure 3D). Taken together, the CCK8 and colony formation results suggested that CD73 overexpression could enhance A549 cells viability, while have no effect on stemness of A549 cells. Furthermore, flow cytometry analysis showed that, compared with A549-NC cells, the ratio of G1 phase cells in A549-CD73 were lower (53.40 vs 56.12%; *p* = 0.015), while, the ratio of S phase cells were higher with no statistical differences (33.02 vs 30.59%; *p* = 0.055), which might be involved in the promoted effect of CD73 on cells proliferation (Supplementary Figure S2). Furthermore, by using scratch assay and transwell assay, we found that CD73 overexpression enhanced migration and invasion ability of A549 cells (Figures 3E,F). These results suggested that the potential function of high CD73 expression in LUAD progression and metastasis. Notably, 100 μM APCP (a specific inhibitor of CD73 enzymatic activity. APCP do not affect the CD73 expression) treatment have no significant influence on proliferation and migration of A549-CD73 (Supplementary Figure S3), which suggested that CD73 might promote LUAD cancer cells proliferation in a enzymatic activity independent manner.

Furthermore, to investigate the effect of CD73 overexpression on LUSC cells, CD73 overexpression cell model was constructed, which named as NCI-H520-CD73 (Supplementary Figures S4A,B). As shown in Supplementary Figures S4C,D there were no significant effect of CD73 overexpression on NCI-H520 cells proliferation and migration. Notably, there were no significant difference of cell cycle between NCI-H520-CD73 and NCI-H520-NC cells (Supplementary Figure S4E). In addition, CD73 overexpression did not affect the expression levels of epithelial to mesenchymal transition (EMT) markers (Supplementary Figure S4F). Taken together, these results suggested the different roles of CD73 in LUAD and LUSC.

### CD73 Overexpression in LUAD Cells Correlated with EMT Progression

**FIGURE 4:**
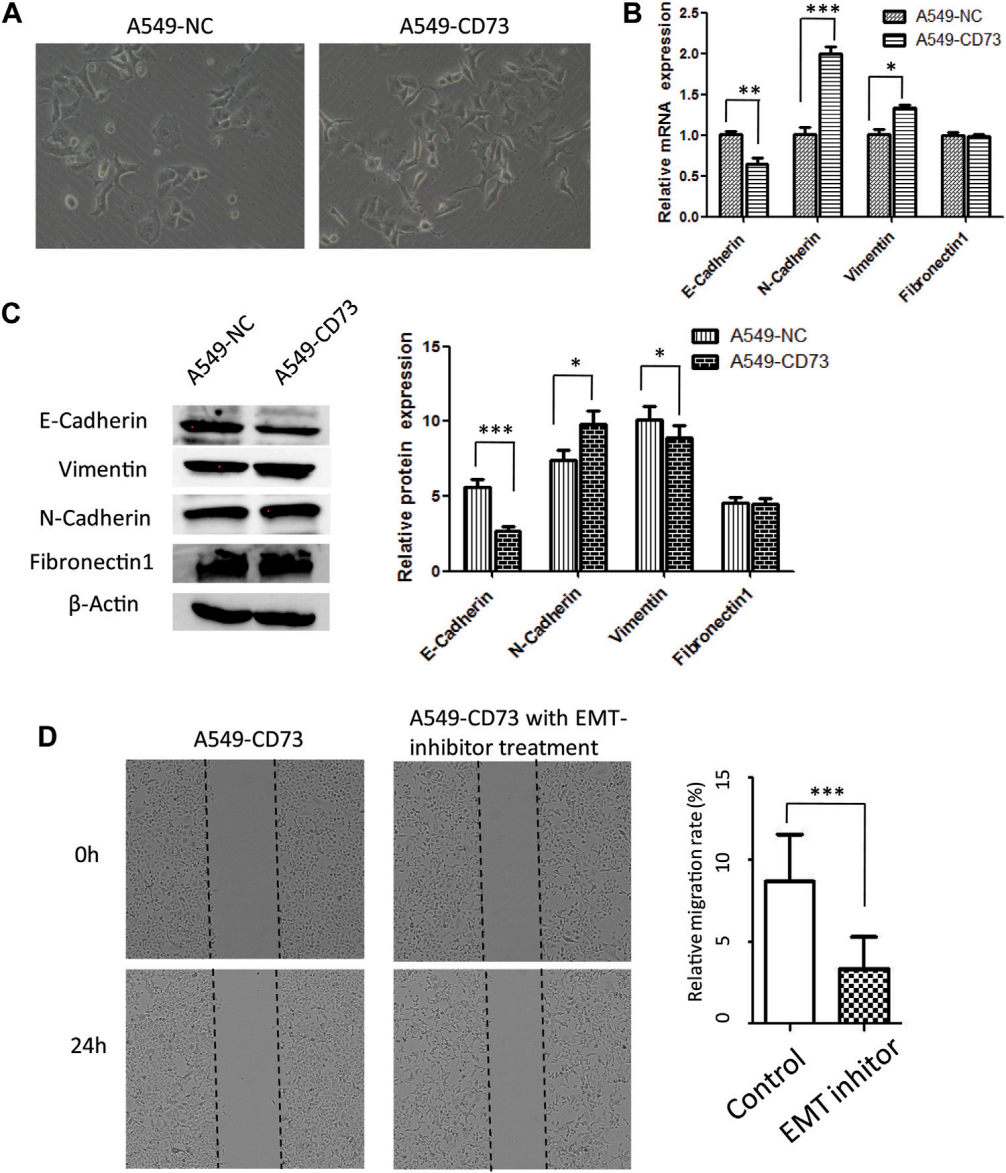
CD73 ovexpression facilitates A549 EMT progression **(A)** The morphology of A549-CD73 and A549-NC. (B, C) EMT markers mRNA **(B)** and protein expression **(C)** levels in CD73 overexpression cells. **(D)** The effect of the EMT-inhibitor treatment on A549-CD73 cells migration.

Epithelial to mesenchymal transition has been considered as an important phenomenon and mechanism during cancer metastasis, which are characterized by loss of epithelial features while increase of mesenchymal features. To further explore the potential mechanism of CD73 overexpression involved in LUAD cells migration. In this study, we investigated the relation between CD73 overexpression and EMT. Our results showed that the morphology of A540-CD73 cells was different from A549-NC cells, A549-CD73 cells become somewhat elongated (Figure 4A). Along with the morphological alterations, the expression level of epithelial marker—E-cadherin was decreased in A549-CD73, while mesenchymal markers (N-cadherin and Vimentin) were increased (Figures 4B,C). Notably, by using EMT-inhibitor (SB431542) treatment, the migration rate of A549-CD73 were significantly decreased (Figure 4D). These results indicated that CD73 overexpression enhanced migration ability of A549 cells via facilitating EMT progression.

## Discussion

Currently, lung cancer is the first leading cause of cancer-related death worldwide. Although there are several target drug have been used in clinical therapy of lung cancer or is in clinical trials, such as EGFR-target TKI (Tyrosine Kinase Inhibitor), ALK-target TKI and VEGF-target Bevacizumab, etc (Fang et al., 2021; Takeyasu et al., 2021; Yu et al., 2021) However, because of the strong heterogeneity, drug resistance and prone to metastasis of lung cancer, the mortality of lung cancer remain high. There is still an urgent need to identify novel therapeutic target and prognostic markers for lung cancer patients.

CD73 has been considered play an important role during cancer progression (Zhi et al., 2012; Griesing et al., 2021). In this study, based on GEPIA2 and GEO database analysis, we observed that the mRNA expression levels of CD73 in LUAD tumor tissues were significantly higher than that in LUSC and non-tumor normal tissues. Notably, GEPIA2 and GEO datasets showed that there were no significant difference of CD73 expression levels between LUSC and normal control tissues. These data suggested that the potential different roles of CD73 involved in LUAD and LUSC progression.

For prognosis, Jiang, T et al.’s study based on meta-analyses have showed the different prognostic value of CD73 were difference among different types of cancers (Jiang., et al., 2018). In this study, based on TCGA and GEO database, high CD73 expression was correlated to poorer OS in LUSC patients. However, via GEPIA2 and KM plotter analysis, we observed the contrary conclusion of the prognostic value of CD73 in LUAD patients, which might be due to different source data for GEPIA2 and KM plotter. And moreover, among different GEO datasets, the association between CD73 expression and LUAD OS were different (HR < 1 in four datasets; HR > 1 in three datasets). Notably, among these datasets, the correlation between CD73 and poor OS with statistical significance were only found in GSE3141 (HR > 1), While there were no statistical significance in other datasets. This result was more likely to support that high CD73 expression as a poor prognostic marker in LUAD patients. Notably, Inoue, Y et al.’s study reported that high CD73 expression was an independent unfavorable prognostic marker in LUAD (Inoue et al., 2017). However, Zeng, Z et al.’s study reported that CD73 were associated with favorable prognosis in LUAD (Zeng et al., 2020). Thus, the prognostic value of CD73 in LUAD still needed further studies.

To elucidate the biological involvement of CD73 in lung cancer progression, we investigate the effect of CD73 overexpression on LUAD cell-A549 and LUSC cell-NCI-H520 proliferation and migration. By conducting cell experiments *in vitro*, we observed the facilitating effect of CD73 overexpression on proliferation and migration ability of A549 cells. However, there were no significant effect of CD73 overexpression on NCI-H520 cells proliferation and migration. These data suggested that the potential different roles of CD73 involved in LUAD and LUSC progression. Interestingly, APCP treatment could not reversed the promotive effect of CD73 overexpression on A549 cells, which indicated the non-enzymatic function of CD73 play an important role in LUAD cancer progression and metastasis. EMT plays an important role in facilitating tumor metastasis. Our study showed that the increased migration in CD73 overexpression A549 cells may be associated with enhancement of EMT process, which was suggested by the differential expression of EMT markers (i.e. E-cadherin, N-cadherin, Vimentin, etc) between A549-CD73 and A549-NC cells. In addition, the EMT inhibitor-SB431542 (Ojima et al., 2020) decrease the A549-CD73 cells migration, which further indicated the relation between CD73 overexpression and EMT progression. Consistently, previous studies have reported the effect of CD73 overexpression on facilitating EMT in other types of tumor, such as gastric cancer, triple-negative breast cancer, ovarian cancer, etc (Lupia et al., 2018; Ma et al., 2019; Xu et al., 2020; Petruk et al., 2021). Taken together, these results indicated a potential correlation between higher CD73 expression and tumor metastatic tendency. Further studies needed to verify the relationship between CD73 and EMT process *in vivo*.

CCK8 and colony formation experiments indicated the different cellular biological behaviors. CCK8 results indicated the change of cell viability, while colony formation results indicated The proportion of subsets of cells with colony-forming capacity, usually tumor stem cells or cells with higher malignant proliferative capacity. Thus, in this study, the CCK8 and colony formation results suggested that CD73 overexpression could enhance A549 cells viability, while have no effect on stemness of A549 cells.

Both enzymatic and non-enzymatic functions of CD73 play important roles in cancer progression. First, for enzymatic function, CD73 is a rate-limiting enzyme in the production of extracellular adenosine. High concentration of CD73-generated adenosine in microenvironment is an anti-inflammatory agent, which prevents excess inflammatory reactions and has been shown to be involved in tumor immunity escapes (Chen et al., 2020; Ishii et al., 2020). Thus, CD73-adenosine axis have been considered as a further immune checkpoint to exploit target treatment. Several clinical trials of anti-CD73 and anti-adenosine-receptor strategies are currently ongoing (Leone and Emens, 2018; Passarelli et al., 2020). In addition, CD73 also exerted its functions in a enzymetic-independent way, which was suggested by the phenomenon that enzymetic activity blocked by APCP treatment does not completely reverse the effect of CD73 overexpression (Gao et al., 2017) Previous studies have shown that CD73 was positively with abnormal EGFR and PI3K/AKT signaling, which is the factors and hallmarks of tumor progression (Zhi et al., 2012; Ma et al., 2019). Notably, both enzymatic and non-enzymatic function were reported to be involved in how CD73 triggered AKT activation. The regulatory network of CD73 in tumor cells should be kept on studying.

In conclusion, our current study suggests that CD73 might be an prognostic marker for LUSC patients. Further studies needed to determine whether the high expression of CD73 is detrimental or beneficial for LUAD patients. And moreover, the effect of CD73 overexpression on lung cancer progression are likely mediated by facilitating tumor cells malignant behavior. These data supports CD73 as a therapeutic target for lung cancer treatment.Zeng et al. (2020).

## Data Availability

The original contributions presented in the study are included in the article/Supplementary Material, further inquiries can be directed to the corresponding author.

## References

[B1] Allard B. Allard D. Buisseret L. Stagg J. (2020). The Adenosine Pathway in Immuno-Oncology. Nat. Rev. Clin. Oncol. 17 (10), 611–629. 10.1038/s41571-020-0382-2 32514148

[B2] Boison D. Yegutkin G. G. (2019). Adenosine Metabolism: Emerging Concepts for Cancer Therapy. Cancer Cell 36 (6), 582–596. 10.1016/j.ccell.2019.10.007 31821783 PMC7224341

[B3] Botling J. Edlund K. Lohr M. Hellwig B. Holmberg L. Lambe M. (2013). Biomarker Discovery in Non-small Cell Lung Cancer: Integrating Gene Expression Profiling, Meta-Analysis, and Tissue Microarray Validation. Clin. Cancer Res. 19 (1), 194–204. 10.1158/1078-0432.CCR-12-1139 23032747

[B4] Brouwer-Visser J. Cheng W.-Y. Bauer-Mehren A. Maisel D. Lechner K. Andersson E. (2018). Regulatory T-Cell Genes Drive Altered Immune Microenvironment in Adult Solid Cancers and Allow for Immune Contextual Patient Subtyping. Cancer Epidemiol. Biomarkers Prev. 27 (1), 103–112. 10.1158/1055-9965.EPI-17-0461 29133367

[B5] Chen Q. Pu N. Yin H. Zhang J. Zhao G. Lou W. (2020). CD73 Acts as a Prognostic Biomarker and Promotes Progression and Immune Escape in Pancreatic Cancer. J. Cel Mol Med 24 (15), 8674–8686. 10.1111/jcmm.15500 PMC7412695 32643277

[B6] Fang L. Zhao W. Ye B. Chen D. (2021). Combination of Immune Checkpoint Inhibitors and Anti-angiogenic Agents in Brain Metastases from Non-small Cell Lung Cancer. Front. Oncol. 11, 670313. 10.3389/fonc.2021.670313 34017689 PMC8130929

[B7] Gao Z.-w. Dong K. Zhang H.-z. (2014). The Roles of CD73 in Cancer. Biomed. Res. Int. 2014, 1–9. 10.1155/2014/460654 PMC4121992 25126561

[B8] Gao Z.-w. Wang H.-p. Lin F. Wang X. Long M. Zhang H.-z. (2017). CD73 Promotes Proliferation and Migration of Human Cervical Cancer Cells Independent of its Enzyme Activity. BMC Cancer 17 (1), 135. 10.1186/s12885-017-3128-5 28202050 PMC5311855

[B9] Gao Z. W. Wang H. P. Dong K. Lin F. Wang X. Zhang H. Z. (2016). Adenosine Inhibits Migration, Invasion and Induces Apoptosis of Human Cervical Cancer Cells. neo 63 (2), 201–207. 10.4149/204_150723N407 26774140

[B10] Griesing S. Liao B.-C. Yang J. C.-H. (2021). CD73 Is Regulated by the EGFR-ERK Signaling Pathway in Non-small Cell Lung Cancer. Anticancer Res. 41 (3), 1231–1242. 10.21873/anticanres.14880 33788714

[B11] Inoue Y. Yoshimura K. Kurabe N. Kahyo T. Kawase A. Tanahashi M. (2017). Prognostic Impact of CD73 and A2A Adenosine Receptor Expression in Non-small-cell Lung Cancer. Oncotarget, 8(5), 8738–8751. 10.18632/oncotarget.14434 28060732 PMC5352437

[B12] Ishii H. Azuma K. Kawahara A. Kinoshita T. Matsuo N. Naito Y. (2020). Predictive Value of CD73 Expression for the Efficacy of Immune Checkpoint Inhibitors in NSCLC. Thorac. Cancer 11 (4), 950–955. 10.1111/1759-7714.13346 32061060 PMC7113063

[B13] Jabs V. Edlund K. König H. Grinberg M. Madjar K. Rahnenführer J. (2017). Integrative Analysis of Genome-wide Gene Copy Number Changes and Gene Expression in Non-small Cell Lung Cancer. PLoS One 12 (11), e0187246. 10.1371/journal.pone.0187246 29112949 PMC5675410

[B14] Jiang T. Xu X. Qiao M. Li X. Zhao C. Zhou F. (2018). Comprehensive Evaluation of NT5E/CD73 Expression and its Prognostic Significance in Distinct Types of Cancers. BMC cancer, 18(1), 267. 10.1186/s12885-018-4073-7 29514610 PMC5842577

[B15] Leone R. D. Emens L. A. (2018). Targeting Adenosine for Cancer Immunotherapy. J. Immunotherapy Cancer 6 (1), 57. 10.1186/s40425-018-0360-8 PMC6006764 29914571

[B16] Lin J. Marquardt G. Mullapudi N. Wang T. Han W. Shi M. (2014). Lung Cancer Transcriptomes Refined with Laser Capture Microdissection. Am. J. Pathol. 184 (11), 2868–2884. 10.1016/j.ajpath.2014.06.028 25128906 PMC4215031

[B17] Lohr M. Hellwig B. Edlund K. Mattsson J. S. M. Botling J. Schmidt M. (2015). Identification of Sample Annotation Errors in Gene Expression Datasets. Arch. Toxicol. 89 (12), 2265–2272. 10.1007/s00204-015-1632-4 26608184 PMC4673097

[B18] Lupia M. Angiolini F. Bertalot G. Freddi S. Sachsenmeier K. F. Chisci E. (2018). CD73 Regulates Stemness and Epithelial-Mesenchymal Transition in Ovarian Cancer-Initiating Cells. Stem Cel Rep. 10 (4), 1412–1425. 10.1016/j.stemcr.2018.02.009 PMC5998305 29551673

[B19] Ma X.-L. Shen M.-N. Hu B. Wang B.-L. Yang W.-J. Lv L.-H. (2019). CD73 Promotes Hepatocellular Carcinoma Progression and Metastasis via Activating PI3K/AKT Signaling by Inducing Rap1-Mediated Membrane Localization of P110β and Predicts Poor Prognosis. J. Hematol. Oncol. 12 (1), 37. 10.1186/s13045-019-0724-7 30971294 PMC6458749

[B20] Menyhárt O. Nagy Á. Győrffy B. (2018). Determining Consistent Prognostic Biomarkers of Overall Survival and Vascular Invasion in Hepatocellular Carcinoma. R. Soc. Open Sci. 5 (12), 181006. 10.1098/rsos.181006 30662724 PMC6304123

[B21] Moreno Leon L. Gautier M. Allan R. Ilié M. Nottet N. Pons N. (2019). The Nuclear Hypoxia-Regulated NLUCAT1 Long Non-coding RNA Contributes to an Aggressive Phenotype in Lung Adenocarcinoma through Regulation of Oxidative Stress. Oncogene 38 (46), 7146–7165. 10.1038/s41388-019-0935-y 31417181

[B22] Ojima T. Kawami M. Yumoto R. Takano M. (2020). Differential Mechanisms Underlying Methotrexate-Induced Cell Death and Epithelial-Mesenchymal Transition in A549 Cells. Toxicol. Res. 37 (3), 293–300. 10.1007/s43188-020-00067-w 34295794 PMC8249484

[B23] Passarelli A. Aieta M. Sgambato A. Gridelli C. (2020). Targeting Immunometabolism Mediated by CD73 Pathway in EGFR-Mutated Non-small Cell Lung Cancer: A New Hope for Overcoming Immune Resistance. Front. Immunol. 11, 1479. 10.3389/fimmu.2020.01479 32760402 PMC7371983

[B24] Petruk N. Tuominen S. Åkerfelt M. Mattsson J. Sandholm J. Nees M. (2021). CD73 Facilitates EMT Progression and Promotes Lung Metastases in Triple-Negative Breast Cancer. Sci. Rep. 11 (1), 6035. 10.1038/s41598-021-85379-z 33727591 PMC7966763

[B25] Sadej R. Skladanowski A. C. (2012). Dual, Enzymatic and Non-enzymatic, Function of Ecto-5'-Nucleotidase (eN, CD73) in Migration and Invasion of A375 Melanoma Cells. Acta Biochim. Pol. 59 (4), 647–652. 10.18388/abp.2012_2105 23162807

[B26] Selamat S. A. Chung B. S. Girard L. Zhang W. Zhang Y. Campan M. (2012). Genome-scale Analysis of DNA Methylation in Lung Adenocarcinoma and Integration with mRNA Expression. Genome Res. 22 (7), 1197–1211. 10.1101/gr.132662.111 22613842 PMC3396362

[B27] Siegel R. L. Miller K. D. Jemal A. (2019). Cancer Statistics, 2019. CA A. Cancer J. Clin. 69 (1), 7–34. 10.3322/caac.21551 30620402

[B28] Takeyasu Y. Okuma H. S. Kojima Y. Nishikawa T. Tanioka M. Sudo K. (2021). Impact of ALK Inhibitors in Patients with ALK-Rearranged Nonlung Solid Tumors. JCO Precis Oncol. 5, 00383. 10.1200/PO.20.00383 PMC8140781 34036223

[B29] Tang Z. Li C. Kang B. Gao G. Li C. Zhang Z. (2017). GEPIA: a Web Server for Cancer and normal Gene Expression Profiling and Interactive Analyses. Nucleic Acids Res. 45 (W1), W98–W102. 10.1093/nar/gkx247 28407145 PMC5570223

[B30] Testa U. Castelli G. Pelosi E. (2018). Lung Cancers: Molecular Characterization, Clonal Heterogeneity and Evolution, and Cancer Stem Cells. Cancers 10 (8), 248. 10.3390/cancers10080248 PMC6116004 30060526

[B31] Tomczak K. Czerwińska P. Wiznerowicz M. (2015). Review the Cancer Genome Atlas (TCGA): an Immeasurable Source of Knowledge. wo 1A (1A), 68–77. 10.5114/wo.2014.47136 PMC4322527 25691825

[B32] Turcotte M. Allard D. Mittal D. Bareche Y. Buisseret L. José V. (2017). CD73 Promotes Resistance to HER2/ErbB2 Antibody Therapy. Cancer Res. 77 (20), 5652–5663. 10.1158/0008-5472.CAN-17-0707 28855210

[B33] Turiello R. Pinto A. Morello S. (2020). CD73: A Promising Biomarker in Cancer Patients. Front. Pharmacol. 11, 609931. 10.3389/fphar.2020.609931 33364969 PMC7751688

[B34] Xu Z. Gu C. Yao X. Guo W. Wang H. Lin T. (2020). CD73 Promotes Tumor Metastasis by Modulating RICS/RhoA Signaling and EMT in Gastric Cancer. Cell Death Dis 11 (3), 202. 10.1038/s41419-020-2403-6 32205841 PMC7089986

[B35] Yang H. Yao F. Davis P. F. Tan S. T. Hall S. R. R. (2021). CD73, Tumor Plasticity and Immune Evasion in Solid Cancers. Cancers 13 (2), 177. 10.3390/cancers13020177 PMC7825701 33430239

[B36] Yu X. Dong Z. Wang W. Mao S. Pan Y. Liu Y. (2021). Adenocarcinoma of High-Grade Patterns Associated with Distinct Outcome of First-Line Chemotherapy or EGFR-TKIs in Patients of Relapsed Lung Cancer. Cmar 13, 3981–3990. 10.2147/CMAR.S302545 PMC8139732 34040439

[B37] Zeng Z. Yang F. Wang Y. Zhao H. Wei F. Zhang P. (2020). Significantly Different Immunological Score in Lung Adenocarcinoma and Squamous Cell Carcinoma and a Proposal for a New Immune Staging System. Oncoimmunology, 9(1), 1828538. 10.1080/2162402X.2020.1828538 33101777 PMC7553570

[B38] Zhi X. Wang Y. Yu J. Yu J. Zhang L. Yin L. (2012). Potential Prognostic Biomarker CD73 Regulates Epidermal Growth Factor Receptor Expression in Human Breast Cancer. IUBMB Life 64 (11), 911–920. 10.1002/iub.1086 23086814

[B39] Zimmermann H. Zebisch M. Sträter N. (2012). Cellular Function and Molecular Structure of Ecto-Nucleotidases. Purinergic Signal. 8 (3), 437–502. 10.1007/s11302-012-9309-4 22555564 PMC3360096

