# Supplementary figures and images for: CD73 Severed as a Potential Prognostic Marker and Promote Lung Cancer Cells Migration via Enhancing EMT Progression
Source: Front Genet. 2021 Nov 17;12:728200. doi: 10.3389/fgene.2021.728200 (PMC8635862; doi:10.3389/fgene.2021.728200)

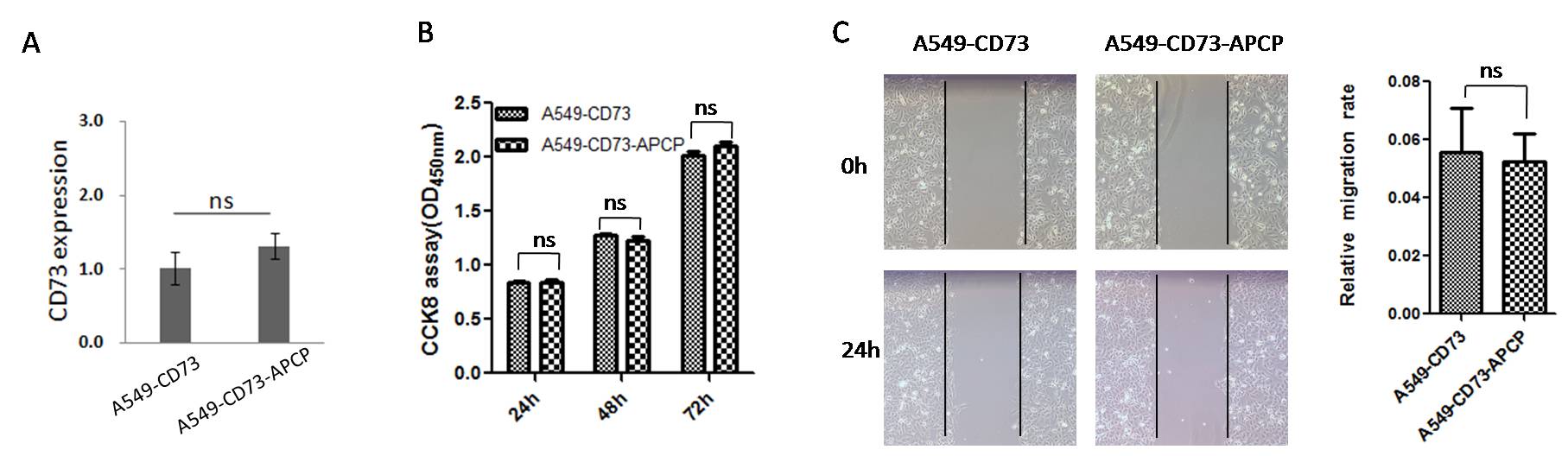

Supplement: Supplementary file 1 [file Image3.JPEG]

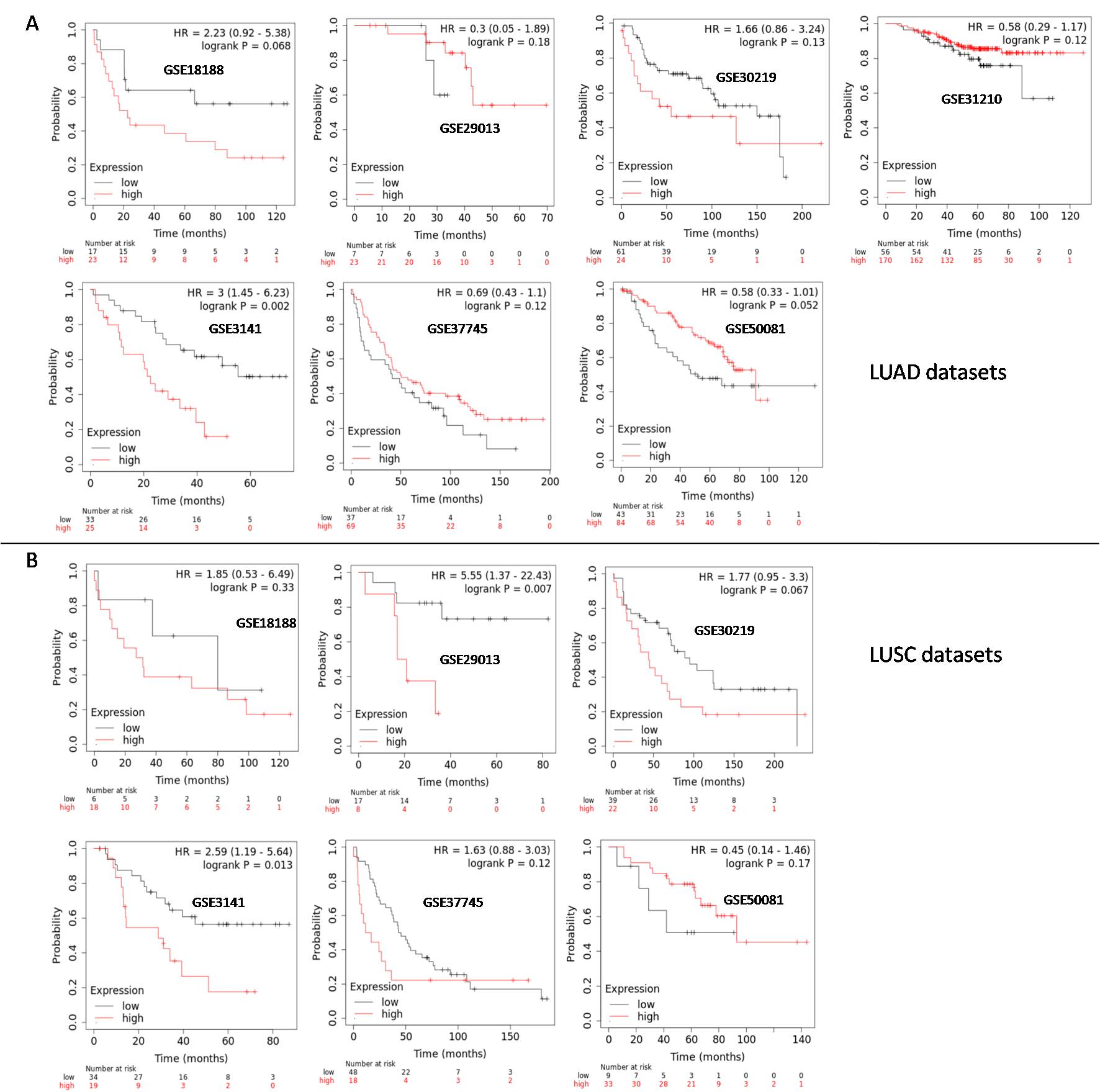

Supplement: Supplementary file 2 [file Image1.JPEG]

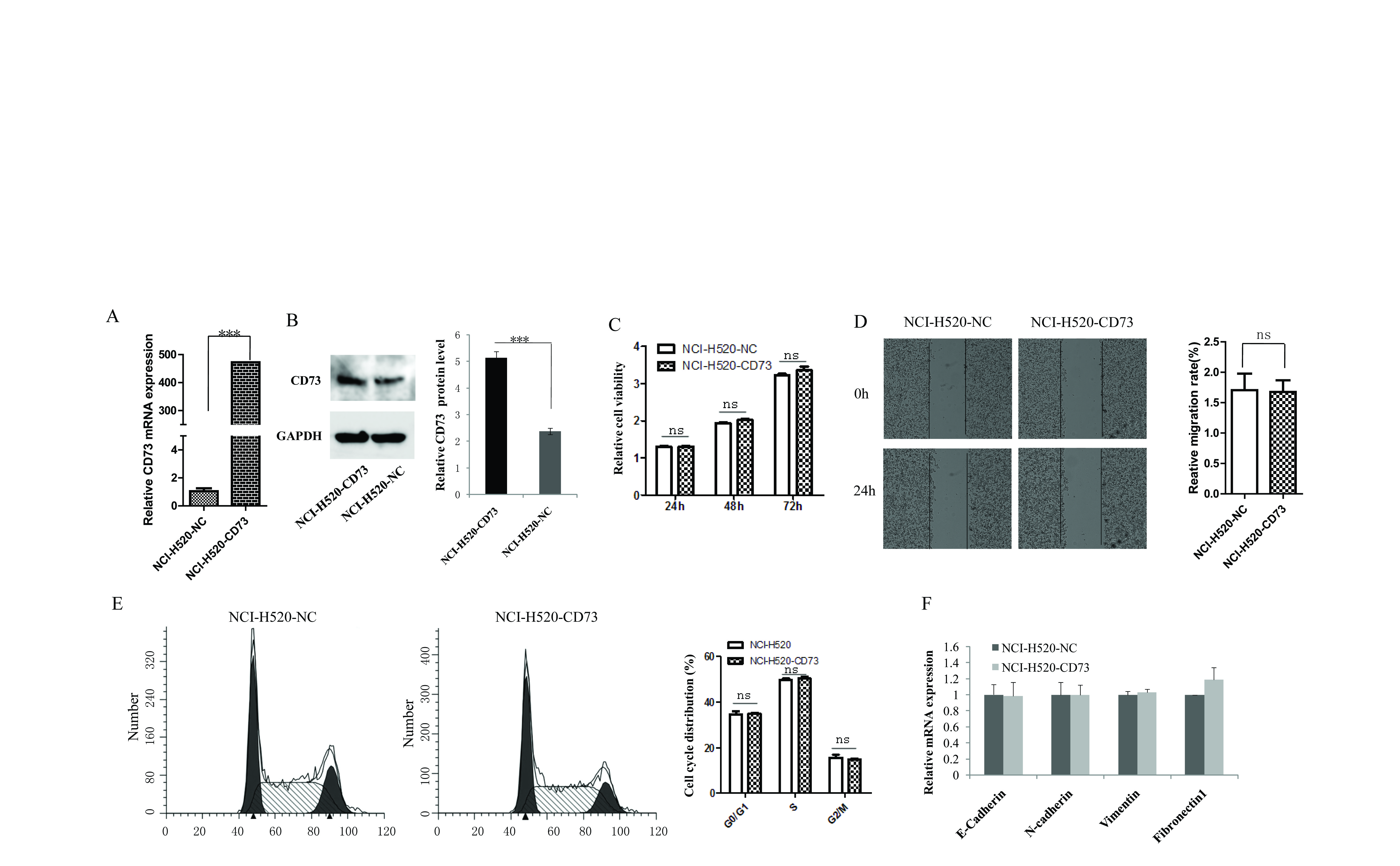

Supplement: Supplementary file 3 [file Image4.JPEG]

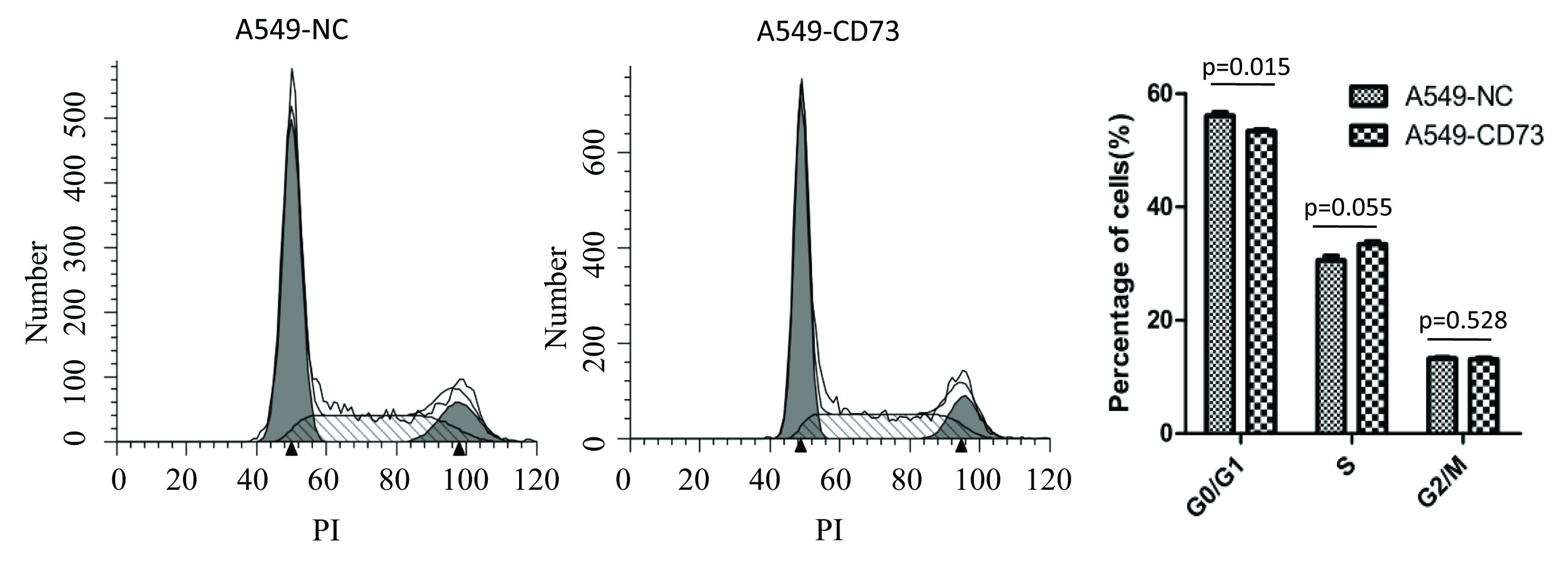

Supplement: Supplementary file 5 [file Image2.JPEG]
