# Supplementary material for: CD73 Severed as a Potential Prognostic Marker and Promote Lung Cancer Cells Migration via Enhancing EMT Progression
Source: Front Genet. 2021 Nov 17;12:728200. doi: 10.3389/fgene.2021.728200 (PMC8635862; doi:10.3389/fgene.2021.728200)
Supplement: Supplementary file 4 [file DataSheet1.ZIP › flow cytometry gating strategise.docx]

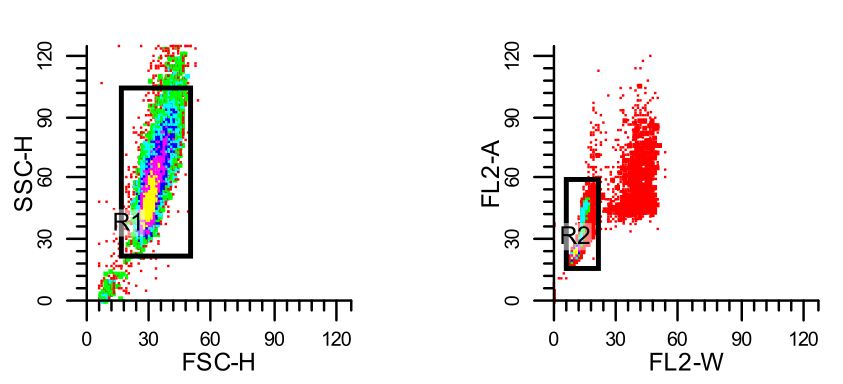


gating strategies

1. Cell debris removal: When data was obtained by flow cytometry, point figure of forward scattering (FSC) and side scattering (SSC) were established, and the FSC threshold was set as 52. Cell group R1 was circled in the point figure of FSC VS SSC during analysis.

2 Remove adhesion cells. using the characteristics of adhesion cells get wider, The population with increased FL2-W value, which is the characteristic of adhesion cells, was discarded in the analysis.
